# Probe-Based Confocal Laser Endomicroscopy for Imaging TRAIL-Expressing Mesenchymal Stem Cells to Monitor Colon Xenograft Tumors *In Vivo*

**DOI:** 10.1371/journal.pone.0162700

**Published:** 2016-09-12

**Authors:** Zhen Zhang, Ming Li, Feixue Chen, Lixiang Li, Jun Liu, Zhen Li, Rui Ji, Xiuli Zuo, Yanqing Li

**Affiliations:** 1 Department of Gastroenterology, Qilu Hospital, Shandong University, Jinan, China; 2 Laboratory of Translational Gastroenterology, Qilu Hospital, Shandong University, Jinan, China; Universite de Nantes, FRANCE

## Abstract

**Introduction:**

Mesenchymal stem cells (MSCs) can serve as vehicles for therapeutic genes. However, little is known about MSC behavior *in vivo*. Here, we demonstrated that probe-based confocal laser endomicroscopy (pCLE) can be used to track MSCs *in vivo* and individually monitor tumor necrosis factor (TNF)-related apoptosis-inducing ligand (TRAIL) gene expression within carcinomas.

**Methods:**

Isolated BALB/c nu/nu mice MSCs (MSCs) were characterized and engineered to co-express the TRAIL and enhanced green fluorescent protein (EGFP) genes. The number of MSCs co-expressing EGFP and TRAIL (TRAIL-MSCs) at tumor sites was quantified with pCLE *in vivo*, while their presence was confirmed using immunofluorescence (IF) and quantitative polymerase chain reaction (qPCR). The therapeutic effects of TRAIL-MSCs were evaluated by measuring the volumes and weights of subcutaneous HT29-derived xenograft tumors.

**Results:**

Intravital imaging of the subcutaneous xenograft tumors revealed that BALB/c mice treated with TRAIL-MSCs exhibited specific cellular signals, whereas no specific signals were observed in the control mice. The findings from the pCLE images were consistent with the IF and qPCR results.

**Conclusion:**

The pCLE results indicated that endomicroscopy could effectively quantify injected MSCs that homed to subcutaneous xenograft tumor sites in vivo and correlated well with the therapeutic effects of the TRAIL gene. By applying pCLE for the in vivo monitoring of cellular trafficking, stem cell-based anticancer gene therapeutic approaches might be feasible and attractive options for individualized clinical treatments.

## 1. Introduction

Colon cancer is the world’s fourth leading cause of death due to cancer in males and the third leading cause in females [1,2]. Despite considerable advances in anti-tumor therapies, colorectal carcinoma remains one of the most challenging diseases. In particular, it has a highly invasive nature, which precludes surgical excision and resists a number of antitumor agents [3]. Therefore, strategies for tracking and killing colon cancer cells remain key challenges for colon cancer therapy.

Under certain specific conditions, mesenchymal stem cells (MSCs) are promising cellular vehicles for cancer therapy. They have self-renewing properties and a strong capacity to migrate into inflamed tissues and active tumors [4–9]. Additionally, they not only possess anti-inflammatory, reparative properties but also can efficiently carry and deliver therapeutic genes into specific locations [10–12]. These qualities merit the investigation of engineered MSCs as novel carriers for the delivery of anti-tumor agents to malignancies.

Tumor necrosis factor (TNF)-related apoptosis inducing ligand (TRAIL) is expressed as a type II transmembrane protein and is a member of the TNF superfamily [13]. TRAIL is an appealing anticancer molecule because it induces cancer cell death without affecting healthy cells [14,15]. Recent reports have shown that generating stable modified MSCs to obtain cellular vehicles leads to targeted and consistent TRAIL delivery, suggesting that synergistic antitumor effects may be achieved using combination therapies [16]. However, determining the most appropriate clinical application of TRAIL-expressing MSCs is currently hampered by a lack of knowledge of how these cells behave *in vivo*.

Probe-based confocal laser endomicroscopy (pCLE), a newly invented diagnostic tool, provides real-time optical section images with a cellular resolution similar to that of histology [17]. Recent studies have indicated that endomicroscopic molecular imaging with fluorescent antibodies has the potential to predict therapeutic responses to biological treatment [18]. The endomicroscopy system uses a blue laser that delivers an excitation wavelength of 465 nm and light emission at 505–585 nm, which is compatible with EGFP [19].

The aim of this work was to investigate the feasibility of using pCLE to visualize the homing of TRAIL-MSCs to tumors *in vivo*.

## 2. Materials and Methods

### 2.1 Colon cancer cell line and culture conditions

HT29 cells (HB-8247, ATCC, USA) were maintained in Dulbecco’s Modified Eagle’s Medium (DMEM) with 10% fetal bovine serum (FBS), 100 U/ml penicillin, and 100 mg/ml streptomycin at 37°C with 5% CO_2_. They were passaged every 3–4 d, and the medium was replaced every 2 d. The cells were detached using 0.05% trypsin-ethylenediaminetetraacetic acid (EDTA), washed with phosphate buffered saline (PBS) and cultured in DMEM with 10% FBS for *in vitro* studies or in PBS for *in vivo* studies.

### 2.2 MSC preparation and characterization

MSCs were obtained and cultured from the bone marrow of 3- to 4-week-old female BALB/c nu/nu mice, according to a previously described protocol [20]. The isolated cells were maintained in complete growth medium (MUCMX-90011, Cyagen, Guangzhou) at 37°C with 5% CO_2_. All experiments used cells from passages 4 and 5 [21].

Cultured MSCs were tested for their ability to differentiate into adipogenic, chondrogenic and osteogenic cell lineages, as previously described [22]. This differentiation potential was characterized using optimized differentiation medium (MUCMX-90021, MUCMX-90031, and MUCMX-90041, respectively; Cyagen, Guangzhou). The immunophenotypes of the MSCs were identified using an antibody panel as previously described [23]. The MSCs were labeled with the following antibodies specific for mouse surface antigens: CD29-fluorescein isothiocyanate (FITC), CD45-phycoerythrin (PE), CD90-PE (BioLegend, San Diego, CA, USA), CD34-FITC and CD44-PE (eBioscience Inc., San Diego, CA, USA). The data were measured using fluorescence-activated cell sorting (FACS).

### 2.3 Adenoviral infection

At passage 2, MSCs were transduced with the CMV-EGFP-EF-1a-TRAIL lentiviral vector (GenePharma, Shanghai) at a multiplicity of infection (MOI) of 100 in complete growth medium. Then, 5 μg/ml polybrene (Sigma, Shanghai) was added to this medium to assist the uptake of viral particles, as previously described [24]. After 48h, the infection efficiency and fluorescence intensity of GFP-positive cells were confirmed via FACS and inverted fluorescence microscopy.

### 2.4 Tumor-bearing mice and cell injection

To further explore the feasibility of intravital molecular imaging of MSCs, a tumor-bearing mouse model was necessary. Four-week-old BALB/c nu/nu mice weighing 15–20 g were maintained under specific pathogen-free conditions and used in accordance with institutional guidelines under approved protocols. Suspensions of 2×10^6^ tumor cells in 100 ml of PBS were administered at a site above the right flank of each mouse. Approximately 1 week after injection, when the tumor size reached 4 to 6 mm in diameter, TRAIL-MSCs (5×10^6^ cells in suspension in 100 ml of PBS) were injected intravenously into the tail vein of the tumor-bearing mice (n = 5). Negative controls included mice injected with PBS alone (n = 5) and mice injected with nontransduced MSCs at the same dosage (n = 5). MSCs were injected only when the xenografts were fully established and this day was defined as day 0. On days 3, 6, 9, 12, 15 and 18, tumor volumes were calculated by measuring their width and height as previously reported [17]. On day 18, the animals were sacrificed, and the tumors were examined to determine their volume and weight. This study was carried out after permission was granted by the Qilu Hospital Committee on the Care and Use of Animals, and we followed the guidelines for animal studies provided in Animal Research: Reporting *In Vivo* Experiments (ARRIVE) [25].

### 2.5 Macroscopic fluorescence imaging

We aimed to monitor MSC migration *in vivo* using macroscopic fluorescence imaging analysis. According to the procedure described by Goetz et al. [26], mice were administered TRAIL-MSCs (5×10^6^ cells in suspension in 100 ml of PBS, n = 3) as experimental group, while mice were injected with MSCs (n = 3) and mice with EGFP-MSCs (n = 3) at the same dosage as the control groups. These mice were then examined to identify the optimal time point for the targeted imaging of MSCs using a Xenogen IVIS-Spectrum system (Caliper Life Sciences, Hopkinton, MA). The fluorescence signals were acquired in a lateral position using a 465-nm excitation filter.

### 2.6 pCLE imaging

*In vitro*, EGFP-labeled MSCs (EGFP-MSCs) and TRAIL-MSCs were imaged by pCLE using a Cellvizio endomicroscopy system (Mauna Kea Technologies, France). The Cellvizio system had a field of view of 240 μm and an imaging depth of 60 μm below the surface of the tumor. Its lateral resolution was 1–1.5 μm, which enabled the identification of individual labeled cells with dead cell debris containing the labeling material. The MSCs were maintained in 6-well plates, and the endomicroscopy equipment was vertically positioned to image the cells. The acquired data were stored in the form of videos (Cellvizio). After anesthesia was administered, the xenografted tumors were examined by pCLE, which is equivalent to integrated flexible CLE [27]. For *in vivo* pCLE imaging, a handheld confocal probe was first gently placed directly onto the tumor surface and was then dipped into the tumor through a small opening that was made using scissors. All tumor tissue was screened for fluorescence signals. The mice injected with nontransduced MSCs and PBS alone were also evaluated as negative controls. All mucosa was screened with the confocal probe for fluorescence signals by carefully moving the probe across the mucosa while adjusting the imaging plane depth. For each observed neoplastic and normal region, three different regions of interest (ROIs) 20×20 μm in size with the strongest fluorescence signals in the representative image were selected. The mean gray-scale value of the three ROIs was calculated within each image, as previously described in detail [27]: black (0)–white (255). the mice were sacrificed by a chloral hydrate overdose. Surface and inner tissue specimens were gently stripped away from the tumor and collected for further examination.

### 2.7 Histological and immunofluorescence examination

After the imaging procedures, the solitary xenograft tumors were fixed in 4% buffered formalin. Hematoxylin and eosin (H&E) staining was performed on serial sections of 4 μm intervals for histological examination. The surface and inner samples from these samples were frozen and sectioned to detect TRAIL and EGFP expression, as previously described [28]. The sections were blocked with normal goat serum for 30 minutes (Zli-9021, ZSGB-BIO, Beijing) and then incubated simultaneously with a 1:100-diluted polyclonal rabbit anti-TRAIL primary antibody (AB2435, Abcam, U.S.A.) and a 1:20-diluted polyclonal mouse anti-EGFP primary antibody (AB16278, Abcam, U.S.A.). Our detection of TRAIL-MSCs was based on incubation with a FITC-conjugated goat anti-rabbit secondary antibody (ZF0311, ZSGB-BIO, Beijing) and a rhodamine-conjugated goat anti-mouse secondary antibody (ZF0313, ZSGB-BIO, Beijing).

### 2.8 Quantitative polymerase chain reaction

Total RNA was extracted from the surface and inner specimens of tumors treated with TRAIL-MSCs and was then compared with that of specimens from the same regions of negative controls (RNA Prep Pure Tissue Kit, DP431, Tiangen, Beijing). Extracted RNA was subjected to reverse transcription (ReverTra Ace qPCR RT Kit, FSQ-101, TOYOBO, Shuzo, Japan), 500 ng of cDNA was processed for real-time PCR amplification using primers specific for TRAIL, and real-time qPCR was performed using SYBR Green MasterMix (TOYOBO, Shuzo, Japan) with primers on a Roche Light Cycler-480 thermal cycler. The primer sequences specific for TRAIL were as follows: forward: 5′-CGGCTGAGATGGCTATGATGGAGGTCC-3′, reverse: 5′-GCCGAATTCTTAGCCAACTAAAAAGGC-3′. The primer sequences for the control gene, glyceraldehyde 3-phosphate dehydrogenase (GAPDH), were as follows: forward: 5′-CGTGGAAGGACTCATGAC-3′, reverse: 5′-CAATTCGTTGTCATACCAG-3′ (Sangon, Shanghai). Total RNA from non-primed MSCs served as a control [28].

### 2.9 Statistical analysis

Significance was determined with Student’s t test when comparing two groups. Data were analyzed using the statistical software packages Statistical Package for Social Science version 17.0 (SPSS Inc., Chicago, IL, USA) and GraphPad Prism version 4 (GraphPad Software). Data are expressed as the mean ± SD with a 95% confidence interval. Differences were considered significant when *P* was less than 0.05. All of the statistical tests were two-sided. The two-sample t-test was used for the comparison of control vs experimental conditions or the surface and inner site at the same samples.

## 3. Results

### 3.1 Characterization of isolated MSCs

**Fig 1:**
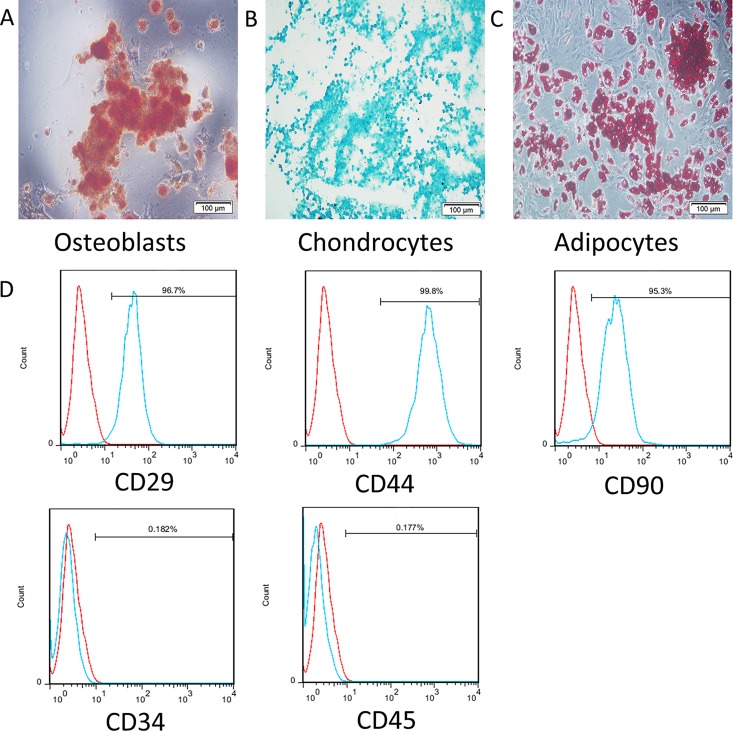
Differential capacities and immunophenotypes of MSCs. (A) Differentiated osteoblasts tested with alkaline phosphatase staining. (B) Differentiated chondrocytes verified by toluidine blue staining. (C) Differentiated adipocytes characterized by oil red O staining. (D) Flow cytometric analysis of surface antigens of bone marrow-derived MSCs from BALB/c nu/nu mice: FITC-CD29, PE-CD44, PE-CD90, FITC-CD34 and PE-CD45.

MSCs were extracted from the bone marrow of female BALB/c nu/nu mice and characterized. The immunophenotyping results and the differential capacity of isolated MSCs were consistent with those that have been previously reported [20]. As plastic-adherent cells, MSCs were maintained *in vitro*. Spindle-shaped cells appeared and gradually predominated within the primary culture. Adipogenic, chondrogenic and osteogenic differentiation assays confirmed that the MSCs were capable of multilineage differentiation (Fig 1A–1C). The flow cytometric analysis of surface markers (Fig 1D) showed that the isolated MSCs were positive for CD29, CD44 and CD90 but negative for CD34 and CD 45, which agreed with the characteristics of MSCs [23].

### 3.2 Adenoviral infection and macroscopic fluorescence imaging

**Fig 2:**
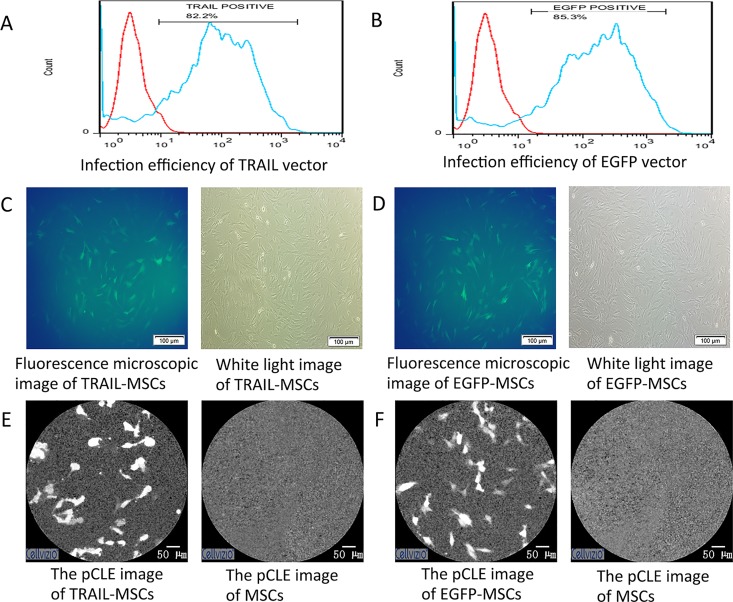
Fluorescence microscopy and pCLE images of MSCs. (A) The infection efficiency of the CMV-eGFP-EF-1a-TRAIL lentiviral vector into MSCs was 81.6% ± 3.1 (n = 3, the data are presented as the mean ± SD). (B) Flow cytometric analysis indicated that 84.6% ± 1.0 (n = 3, the data are presented as the mean ± SD) of the MSCs transduced with the lentiviral vector containing EGFP were GFP positive. (C) Fluorescence microscopy images of TRAIL-MSCs (left) and white light images of TRAIL-MSCs (right). (D) Fluorescence microscopy images of EGFP-MSCs (left) and white light images of TRAIL-MSCs (right). (E) pCLE images of TRAIL-MSCs (left) and nontransduced MSCs (right) *ex vivo*. (F) pCLE images of EGFP-MSCs (left) and nontransduced MSCs (right) *ex vivo*.

**Fig 3:**
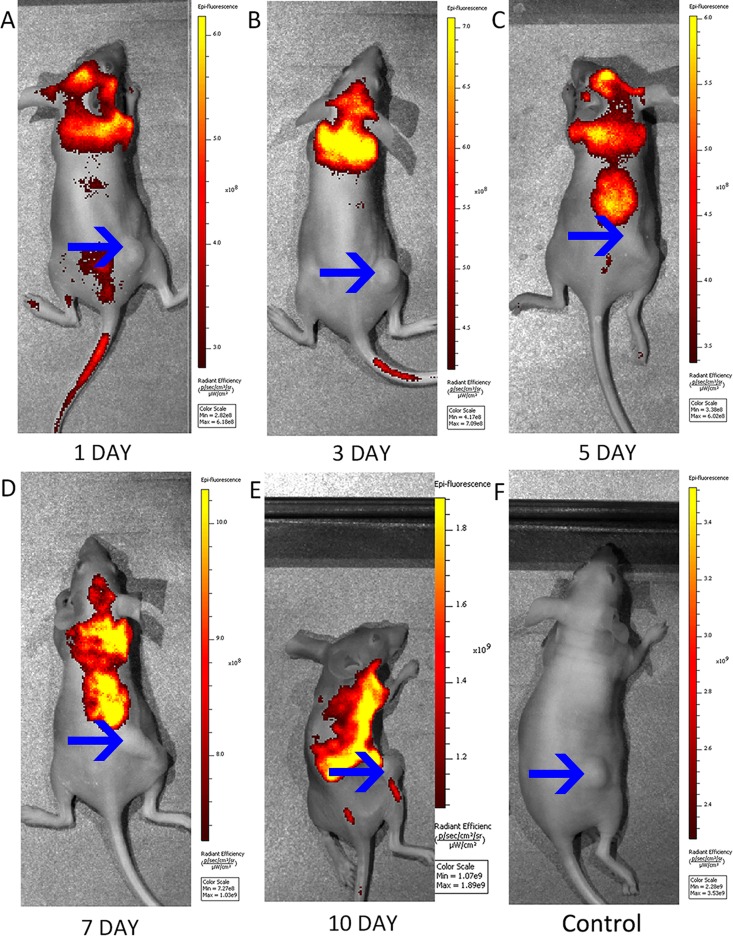
Macroscopic fluorescence imaging of tumor-bearing mice. (A-E) Movement of strong fluorescent signals was observed in subcutaneous xenograft models at 1, 3, 5, 7, and 10 days after the intravenous injection of TRAIL-MSCs (n = 3). The arrows show the tumor locations. (F) No significant fluorescence signals were observed around tumor sites in the mice injected with MSCs (n = 3) as the control group.

The flow cytometric analysis indicated that the infection efficiency of the CMV-EGFP-EF-1a-TRAIL lentiviral vector into the MSCs was 81.6% ± 3.1. Furthermore, 84.6% ± 1.0 of the MSCs transduced with the lentiviral vector containing EGFP were GFP-positive (Fig 2A and 2B). The FACS analysis showed that the intensity of the fluorescence signals of the TRAIL-MSCs was similar to that of the EGFP-MSCs, which correlated well with the fluorescence microscopy and pCLE results (Fig 2C–2F). The ability of MSCs to migrate *in vivo* toward tumor sites was first determined through macroscopic imaging. In a first study of macroscopic fluorescence imaging in tumor-bearing mice, a significant change in signal movement wasf observed in mice treated with TRAIL-MSCs compared with mice treated with nontransduced MSCs (n = 3, Fig 3A–3F). In addition, specific fluorescence signals were observed at the tumor sites 10 days after the injection of TRAIL-MSCs, which was similar with the findings of a previous study [29]. Macroscopic fluorescence imaging indicated potentially the specificity of *in vivo* TRAIL-MSC homing. Our macroscopic fluorescence imaging results showed that the movement of the TRAIL-MSCs and EGFP-MSCs in the xenografts was similar (n = 3, S1 Fig).

### 3.3 Endomicroscopic imaging permits the assessment of engineered MSC fate *in vivo*

**Fig 4:**
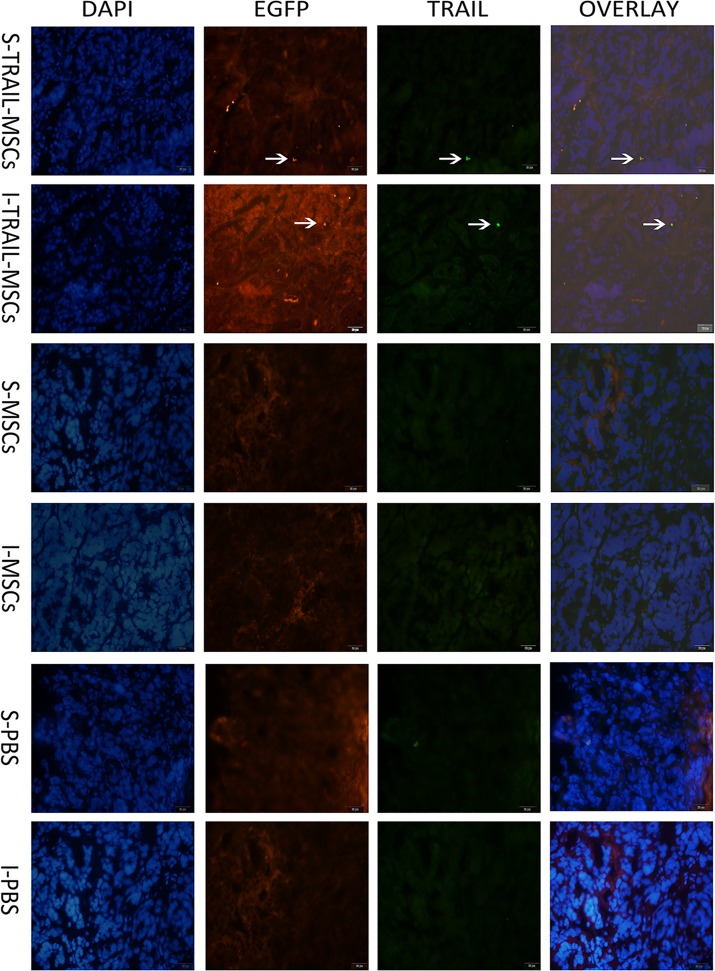
Immunofluorescence results. The immunofluorescence results showed that the fluorescence signal of TRAIL (green) and fluorescence signal of EGFP (red) were tested at the same MSCs in the tumor site. It revealed that isolated double-labeled TRAIL-positive cells were present on the surface (S-TRAIL-MSCs) and inside (I-TRAIL-MSCs) of tumors from the TRAIL-MSCs treated mice, while no double-labeled cells were observed at the same section in samples from mice treated with MSCs (S-MSCs, I-MSCs) or PBS (S-PBS, I-PBS) as the control group. Cell nucleus was stained blue by DAPI. Scale bar = 20 μm.

To confirm these data, as previously described [28], IF was used to detect TRAIL-MSCs in the same tumor sections that were imaged with pCLE at day 18 (Fig 4). TRAIL-MSCs stained positively on the surface of tumor lesions from xenografts in mice, whereas staining was also observed inside the tumors, while no double-labeled cells were observed at the same section in samples from mice treated with MSC or PBS as the control group. Moreover, H&E staining was used to confirm the surface and inner colon tumor sites (S2D Fig).

**Fig 5:**
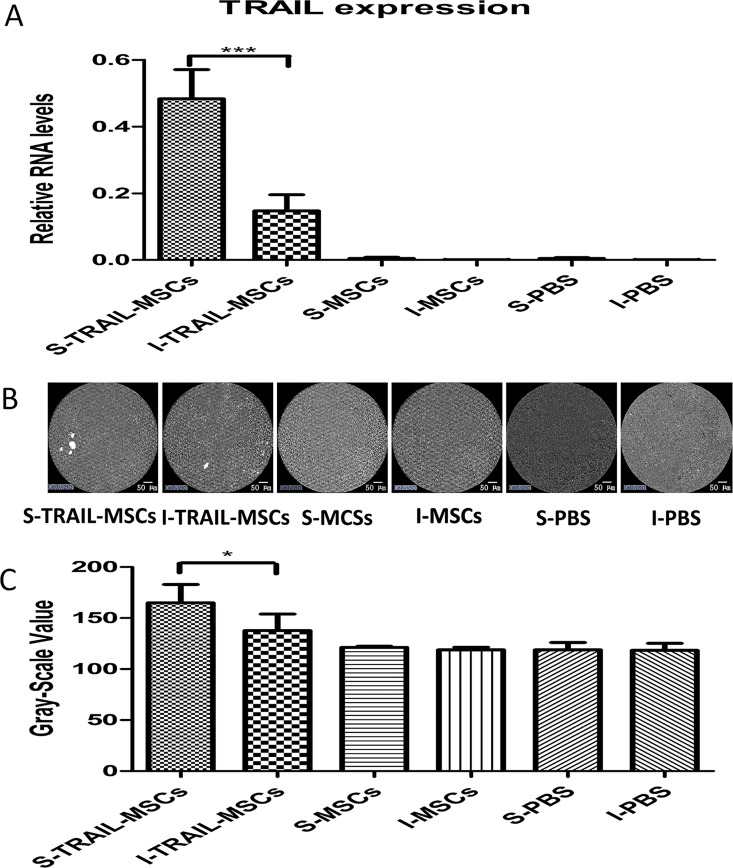
*In vivo* tracing of TRAIL-MSCs. (A) The expression of TRAIL on the surface of tumors from mice treated with TRAIL-MSCs (S-TRAIL-MSCs), the inside of tumors from mice treated with TRAIL-MSCs (I-TRAIL-MSCs), the surface of tumors from mice treated with nontransduced MSCs (S-MSCs) and the inside of tumors from mice treated with nontransduced MSCs (I-MSCs) were evaluated by qPCR. *** *P*<0.001, n = 5. The data are presented as the mean ± SD. (B) Endomicroscopy images of TRAIL-MSC compared with nontransduced MSC and PBS groups localized to the surface and inside of tumors. Scale bar = 50 μm. (C) The mean gray-scale value of ROIs of from TRAIL-MSC, MSC and PBS groups on the surface and inside of tumors. * *P*<0.05, n = 5. The data are presented as the mean ± SD.

We examined the presence of TRAIL expression from transplanted MSCs by qPCR *in vitro*. In general, the expression levels of TRAIL at the surface of tumors obtained via TRAIL-MSC injection were significantly higher than the expression levels of TRAIL inside of the tumors (*P*<0.001, n = 5, Fig 5A), compared with the same sections in MSC or PBS samples as the control.

Based on the time and dosage of TRAIL-MSCs observed by full-body imaging, we used the Cellvizio system to test MSC imaging capability at day 18. After an intravenous injection of TRAIL-MSCs, fluorescent cellular signals could be visualized at the surface of the tumors. The signals detected inside the tumor sites were weaker than those at the surface (P<0.05, n = 5, Fig 5B and 5C). The tumors treated with nontransduced MSCs and PBS alone, which served as the negative controls, demonstrated no specific fluorescence signals using pCLE imaging *in vivo*. The pCLE images of healthy areas taken at distance from tumors in TRAIL-MSCs treated group, compared with the same sections in MSC or PBS samples as the control (S2A–S2C Fig). These results indicated that injected MSCs migrated to the periphery of the tumors, but few cells entered the tumor, suggesting that the main role of MSCs in tumor progression might be related to tumor angiogenesis [30]. The sensitivity of tracing TRAIL-MSCs using pCLE was correlated with the qPCR results. These results may suggest that *in vivo* endomicroscopy is an effective method for tracking stem cells *in vivo*.

### 3.4 TRAIL-MSCs exert anti-tumor effects

**Fig 6:**
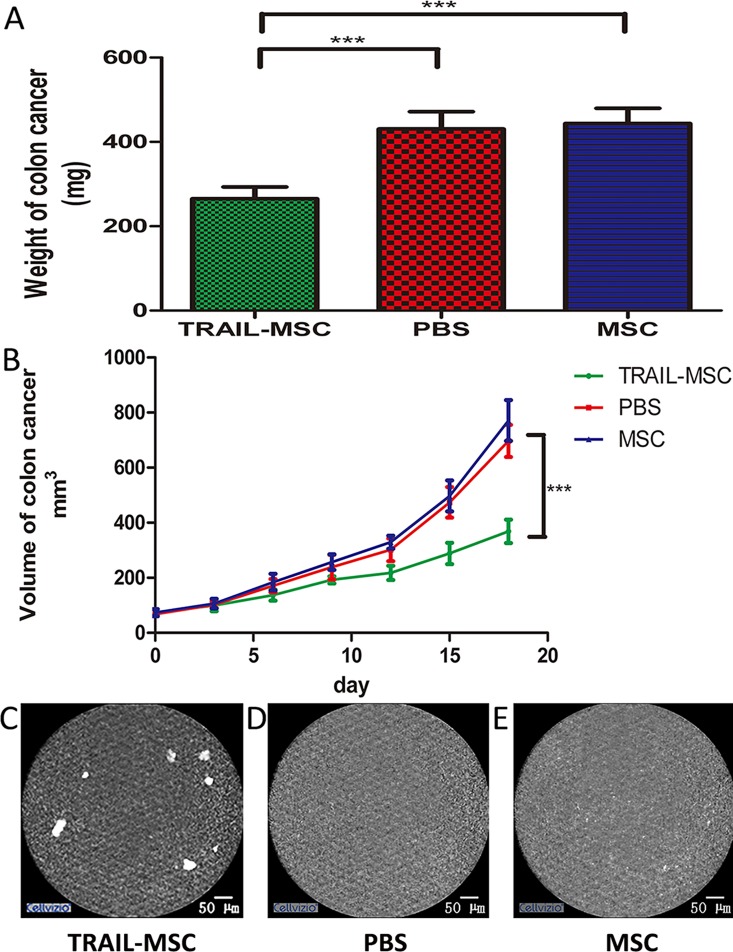
TRAIL-MSCs can be tracked *in vivo* and correspond to tumor growth. (A) Weights and (B) volumes of colon cancer tumors isolated from xenografts in mice after injection with TRAIL-MSCs and a period of 18 days. Volume = length×width^2^/2 [46]. *** *P*<0.001, n = 5. The data are presented as the mean ± SD. (C) Endomicroscopy images of TRAIL-MSCs on the surface of tumors. (D) Mice injected with PBS alone and (E) mice injected with nontransduced MSCs were used as negative controls.

Next, we further investigated whether endomicroscopy images of TRAIL-MSCs *in vivo* had strong correlations with the therapeutic effects of the TRAIL gene on colon cancer xenografts in mice. Such a relationship could streamline the clinical administration of MSCs in the future. The weights and volumes of the tumors derived from mice injected with TRAIL-MSCs were significantly reduced relative to control mice (*P*<0.05, n = 5, Fig 6A and 6B). The specific fluorescence signals in the pCLE images demonstrated that some of the injected TRAIL-MSCs localized to the colorectal neoplasia (Fig 6C), which corresponded with a reduction in tumor growth in the mice treated with TRAIL-MSCs compared with those treated with PBS and nontransduced MSCs. These results suggested that endomicroscopy could be used as a stem cell detection tool *in vivo*.

## 4. Discussion

In this study, for the first time, we demonstrated that the homing of EGFP-labeled TRAIL-MSCs to colon xenograft tumors can be endomicroscopically monitored *in vivo*. Moreover, it is anticipated that pCLE has the potential for analyzing TRAIL-MSCs in the treatment of colon subcutaneous xenograft tumors in the future.

The use of MSCs as vectors for gene therapy is becoming increasingly common. Because MSCs are easy to extract from bone marrow and permit allogeneic transplantation without immunosuppressive drugs due to a lack of significant immunogenicity [31]. However, it is not definitively clear whether MSCs home to multiple tumor types *in vivo*, which restricts the translation of MSC research into clinical applications [32,33]. The effects of recombinant MSCs on tumor development have been shown in different types of cancers such as melanoma, lymphoma, and colon cancer [34–36]. TRAIL-MSCs are known to eliminate tumor growth in cancer models *in vivo* [37], but their effects have not been assessed in clinical trials, likely due to the lack of an effective method to monitor the cells and measure progress.

Currently, there are several methods to track the fluorescence signals of MSCs *in vivo*. Macroscopic fluorescence imaging have been applied as non-invasive methods to track MSC migration and monitor therapeutic efficacy in tumor models [38]. Although this method has become an effective tool and has provided accurate results in pre-clinical studies [39], its clinical utility is hindered by low spatial resolution and poor tissue penetration, making it unfeasible for use in patient trials. Magnetic resonance imaging (MRI) and positron emission-computed tomography (PET) imaging are commonly used in pre-clinical and clinical studies to visualize various tumor and drug interactions. However, radio-labeling methods have some drawbacks, such as exposure to radiation and an inconvenient detection procedure. The utility of MRI to trace superparamagnetic particle-labeled MSCs has been extensively studied [40–42]. Although MRI can reveal the global distribution of MSCs across organs, for clinical use in patients. The limitations of this approach include the possibility of false-positive interpretation of the MRI signals, which may be produced by dead cell debris containing the labeling material [43]. Moreover, MRI cannot not adequately monitor the intestine due to the peristalsis that occurs while imaging 1 frame. [44]. PET imaging can also track MSCs with similar performance. Furthermore, the clinical spatial resolution is only 2–4 mm, which is sufficient to guide additional interventional therapies. Unfortunately, PET imaging is expensive, and PET scans take longer than MRI scans [45]. However, neither of these methods can track stem cells at a cellular resolution.

This study represents the first time that we have used an endoscopic method to track EGFP-labeled MSCs in colon xenograft tumors. There are three major advantages of this approach. First, tracking MSCs via endomicroscopy will help reduce pain and the number of unnecessary biopsies. Second, colon cancer is primarily located in the colorectum, which is close to the anus. It is very handy to get the probe of pCLE reach to the colon cancer through anus. Third, endomicroscopy can rapidly provide ultra-high-resolution images to visualize individual cells. The therapeutic effects on colon cancer observed with intravenous delivery can be correlated with cellular morphology, fluorescence intensity and MSC counts within the tumors, which will be important for TRAIL-MSC-based therapies. Ideally, the administration of MSCs should be individualized according to the specified conditions in each patient [43]. With this method, targeted individualized therapies could be based on the enhanced tracing and accurate location of gene modification vehicles. The combination of fluorescently labeled MSCs and visualization using pCLE represents a novel method for real-time in vivo microscopic imaging of colon cancer models. These properties will enable investigators to evaluate the delivery of high-dose-targeted cancer therapies directly to tumors by employing pCLE. This method is far more practical and financially feasible for patients compared with other radiological approaches, making it a clinically attractive option.

There are some limitations to our method of imaging TRAIL-MSCs in colorectal neoplasia using pCLE. First, only xenograft animal models were evaluated in this study, and surgical orthotopic implantation would provide a more accurate clinical model [47]. Nevertheless, because the primary aim of this study was to evaluate the feasibility of using fluorescence imaging to track TRAIL-MSCs in human colorectal neoplasias, surgical orthotopic implantation was not carried out. Furthermore, the pCLE probe could not easily pass through the anus in the mouse model. Second, the previous study has shown that the resistance of HT29 cell line to TRAIL-MSCs could be achieved in vivo [48]. However, the methods used in that study were fairly different from those in my study. In addition, other researchers have reported that tumor-homing particles efficiently enable the sensitization of tumors to TRAIL with low systemic toxicity [49–50]. Third, although we can infer that the fluorescence signals of TRAIL-MSCs tracked in vivo are closely related to the therapeutic potential of TRAIL-MSCs, it is difficult to detect tumor development using only pCLE. Ideally, such approaches would be combined with white-light endomicroscopy to assess the resultant anti-tumor effects in clinical applications. Additionally, only xenograft animal models were evaluated in this study, and the principles of this approach may not directly correlate to humans. Large sample sizes and more precise administration are needed to overcome the limitations of our study. Further studies evaluating the feasibility and potential benefits of endomicroscopically monitoring MSCs in humans are required.

In conclusion, our study demonstrates, for the first time, that *in vivo* endomicroscopic cellular imaging of TRAIL-MSCs in xenografts with colorectal neoplasia is possible using pCLE. Furthermore, we have extended our experiments to infer the value of endomicroscopy for assessing the adequacy of TRAIL-MSCs for use as a cell-based anti-tumor therapy. Our data revealed that the endomicroscopic tracking of EGFP-labeled TRAIL-MSCs may be of critical importance for monitoring MSC homing effects and establishing individualized stem cell therapies, as well as maximizing the benefits and preventing the shortcomings of the therapeutic use of MSCs.

## Supporting Information

S1 Fig Macroscopic fluorescence imaging of mice injected with EGFP-MSCs. (A-E) The movement of strong fluorescent signals was observed in subcutaneous xenograft models at 1, 3, 5, 7, and 10 days after intravenous injection of EGFP-MSCs (5×10^6^ cells in suspension in 100 ml of PBS, n = 3). The blue arrows show the tumor locations. (F) No significant fluorescence signals were observed around tumor sites in the mice injected with MSCs (n = 3) as the control group.
(DOCX)

S2 Fig The pCLE and Histologic section staining images from colon tumor. (A) The pCLE images of healthy areas taken at distance from tumor in TRAIL-MSCs treated group, compared with the same sections in (B) MSC or (C) PBS samples as the control. (D) H&E staining distinguished the surface (black arrow) and inner (blue arrow) of colon tumor sites. Scale bar = 100 μm.
(DOCX)
