# Supplementary material for: Probe-Based Confocal Laser Endomicroscopy for Imaging TRAIL-Expressing Mesenchymal Stem Cells to Monitor Colon Xenograft Tumors In Vivo
Source: PLoS One. 2016 Sep 12;11(9):e0162700. doi: 10.1371/journal.pone.0162700 (PMC5019474; doi:10.1371/journal.pone.0162700)
Supplement: S1 Fig — (A-E) The movement of strong fluorescent signals was observed in subcutaneous xenograft models at 1, 3, 5, 7, and 10 days after intravenous injection of EGFP-MSCs (5×10^6^ cells in suspension in 100 ml of PBS, n = 3). The blue arrows show the tumor locations. (F) No significant fluorescence signals were observed around tumor sites in the mice injected with MSCs (n = 3) as the control group. (DOCX) [file pone.0162700.s001.docx]

**
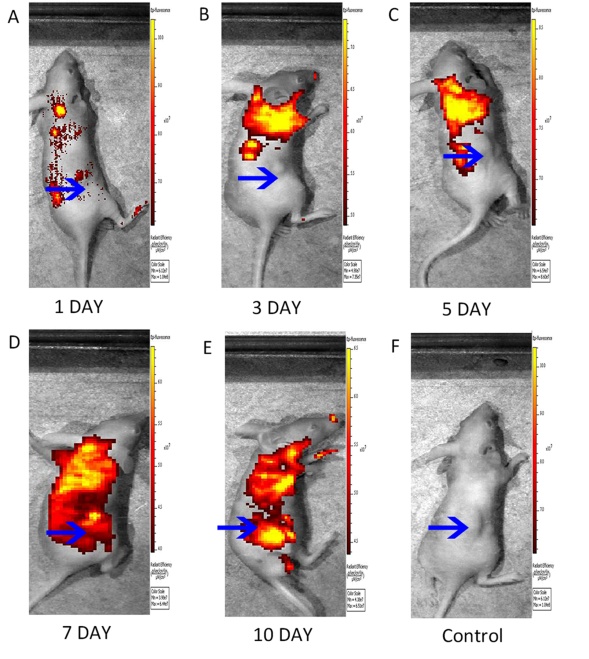
**

**S1 Fig. Macroscopic fluorescence imaging of mice injected with EGFP-MSCs.** (A-E) The movement of strong fluorescent signals was observed in subcutaneous xenograft models at 1, 3, 5, 7, and 10 days after intravenous injection of EGFP-MSCs (5×10^6^ cells in suspension in 100 ml of PBS，n=3). The blue arrows show the tumor locations. (F) No significant fluorescence signals were observed around tumor sites in the mice injected with MSCs (n=3) as the control group.
