# Supplementary material for: Probe-Based Confocal Laser Endomicroscopy for Imaging TRAIL-Expressing Mesenchymal Stem Cells to Monitor Colon Xenograft Tumors In Vivo
Source: PLoS One. 2016 Sep 12;11(9):e0162700. doi: 10.1371/journal.pone.0162700 (PMC5019474; doi:10.1371/journal.pone.0162700)
Supplement: S2 Fig — (A) The pCLE images of healthy areas taken at distance from tumor in TRAIL-MSCs treated group, compared with the same sections in (B) MSC or (C) PBS samples as the control. (D) H&E staining distinguished the surface (black arrow) and inner (blue arrow) of colon tumor sites. Scale bar = 100 μm. (DOCX) [file pone.0162700.s002.docx]

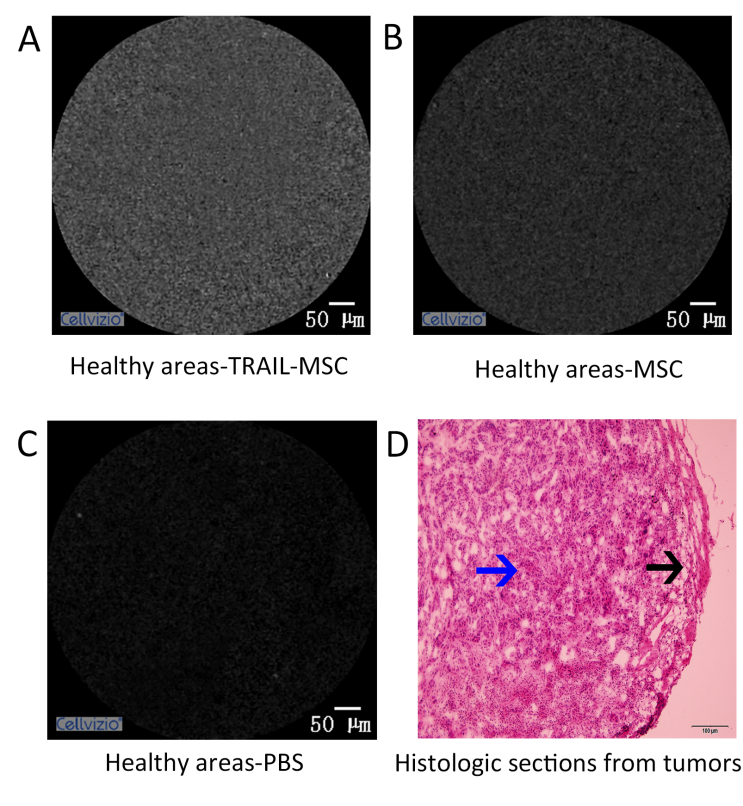


**S2 Fig. The pCLE and Histologic section staining images from colon tumor.** (A) The pCLE images of healthy areas taken at distance from tumor in TRAIL-MSCs treated group, compared with the same sections in (B) MSC or (C) PBS samples as the control. (D) H&E staining distinguished the surface (black arrow) and inner (blue arrow) of colon tumor sites. Scale bar = 100 μm.
